# J-curve relation between daytime nap duration and type 2 diabetes or metabolic syndrome: A dose-response meta-analysis

**DOI:** 10.1038/srep38075

**Published:** 2016-12-02

**Authors:** Tomohide Yamada, Nobuhiro Shojima, Toshimasa Yamauchi, Takashi Kadowaki

**Affiliations:** 1 Department of Diabetes and Metabolic Diseases, Graduate School of Medicine, University of Tokyo, Japan

## Abstract

Adequate sleep is important for good health, but it is not always easy to achieve because of social factors. Daytime napping is widely prevalent around the world. We performed a meta-analysis to investigate the association between napping (or excessive daytime sleepiness: EDS) and the risk of type 2 diabetes or metabolic syndrome, and to quantify the potential dose-response relation using cubic spline models. Electronic databases were searched for articles published up to 2016, with 288,883 Asian and Western subjects. Pooled analysis revealed that a long nap (≥60 min/day) and EDS were each significantly associated with an increased risk of type 2 diabetes versus no nap or no EDS (odds ratio 1.46 (95% CI 1.23–1.74, p < 0.01) for a long nap and 2.00 (1.58–2.53) for EDS). In contrast, a short nap (<60 min/day) was not associated with diabetes (p = 0.75). Dose-response meta-analysis showed a J-curve relation between nap time and the risk of diabetes or metabolic syndrome, with no effect of napping up to about 40 minutes/day, followed by a sharp increase in risk at longer nap times. In summary, longer napping is associated with an increased risk of metabolic disease. Further studies are needed to confirm the benefit of a short nap.

Adequate sleep is important for maintaining good health, along with a balanced diet and exercise. Several recent studies have shown that the relation between the duration of nocturnal sleep and the risk of type 2 diabetes^1^,^2^, CVD^3^, stroke^4^, or all-cause mortality^5^,^6^ is described by a U-shaped curve. These findings suggest that a moderate amount of sleep, neither too short nor too long, promotes good health. However, it is not always easy to obtain sufficient sleep due to the influence of social factors including work. According to the U.S. Centers for Diseases Control and Prevention, one in three American adults do not get enough sleep^7^.

A nap is defined as a short sleep that is typically taken during daylight hours, and the habit of napping is prevalent worldwide. While a daytime nap is usually brief, it can range from a few minutes to several hours. In addition, the frequency varies from occasional naps to several times daily for habitual nappers. Some people take a nap because of excessive daytime drowsiness resulting from a sleep disorder^8^. Similar to the duration of nighttime sleep, several studies have shown that a long daytime nap is positively correlated with cardiovascular disease^9^ and all-cause mortality^10^.

A short nap (<30 min) promotes alertness, reduces sleep deficits, and enhances performance and learning^11^. Some epidemiological studies have even suggested that a short nap decreases the risk of cardiovascular disease^12^ and Alzheimer’s disease^13^.

We recently published a dose-response meta-analysis that identified a J-curve relationship between nap time and cardiovascular disease based on 1.5 million person-years of data^14^. The relative risk of cardiovascular disease was decreased by a short nap (0 to 30 min), followed by a sharp increase at longer nap times. In addition, there was a linear relationship between nap time and all-cause mortality.

Recent epidemiological studies on the relation between daytime napping and diabetes or metabolic syndrome have yielded conflicting results^15^,^16^,^17^,^18^,^19^,^20^. However, these studies showed heterogeneity with respect to sample size, stratification of nap times, nocturnal sleep duration, and other characteristics that could have contributed to different outcomes. It is still unclear whether a dose-response relation exists between daytime napping and the risk of diabetes. Therefore, we performed the present meta-analysis to investigate the association between napping or excessive daytime sleepiness (EDS)^21^,^22^,^23^,^24^ and the risk of diabetes or metabolic syndrome, and we also quantified the potential dose-response relation by using cubic spline models.

## Methods

### Data Sources and Searches

We performed a literature search (up to 31 January 2016) of the Medline, Cochrane Library, and Web of Science databases to identify observational studies examining the association between napping and/or excessive daytime sleepiness and the risk of type 2 diabetes and/or metabolic syndrome. The details of the search terms are shown in Table S1. We supplemented this search by performing a manual search of all the references cited in the articles thus identified.

### Study selection

We selected studies that reported risk estimates for type 2 diabetes and metabolic syndrome in relation to daytime napping and excessive daytime sleepiness in the general population, and that provided point estimates of odds ratio with the 95% confidence interval or standard error for qualitative assessment. We performed a combined meta-analysis of the relation between napping and diabetes or excessive daytime sleepiness and diabetes, because napping and daytime sleepiness have overlapping features and are often handled in the same way clinically by physicians and co-medical staff. We defined the presence of daytime napping on the basis of an affirmative answer to questions such as “Do you take a daytime nap?” or “Do you sleep during the day?” Excessive daytime sleepiness was identified by affirmative answers to questions like “Do you have a problem with sleepiness during the daytime?” We excluded studies on type 1 diabetes. The studies that we selected followed the relevant local rules for ethics and data protection. Studies that did not report risks independently stratified by at least 3 nap time categories (e.g., 0 min, <60 min, and >60 min per day) were excluded from this meta-analysis.

### Data Extraction and Assessment of Study Quality

We extracted information on the characteristics of each study (study name, authors, year of publication, journal, study type, study location, and number of participants and incident cases), the subject characteristics (age, sex, and BMI), the extent of exposure to napping (definition of napping, nap time, and prevalence of napping in each category), the validity of the method used for assessment of napping (and excessive daytime sleepiness), the validity of the method used for assessment of the outcome (diabetes and metabolic syndrome), and the validity of the analytical methods (statistical models, covariates included in the models, and risk estimates for each nap duration category). Some studies reported data separately for men and women, so we treated each of these cohorts as an independent report and extracted data separately. If more than one study covered the same cohort, only the report containing the most comprehensive information was used to avoid analysis of overlapping populations.

To ascertain the validity of the studies, the quality of each report was appraised with reference to the STROBE statement^25^. In addition, the Newcastle-Ottawa Scale for assessing the quality of observational studies in meta-analyses was used to quantify the validity of each study^26^. Only high-quality observational studies with a Newcastle-Ottawa Scale score ≥6 (maximum possible score: 9) were included in this meta-analysis. Two authors (T Yamada and NS) independently confirmed the eligibility of the studies and then extracted and collated the data. Any discrepancies were resolved through discussion.

### Statistical analysis

The pooled odds ratio (OR) and its 95% confidence interval (CI) was employed to assess associations in all of the studies, except for use of the prevalence ratio by Stang *et al*.^15^. Because the incidence of events was not high in their study, the prevalence ratio was considered to be a relatively accurate estimate of the true OR. We pooled all odds ratios by using the DerSimonian-Laird random effects model to compare napping categories and set study weights as equal to the inverse variance of the estimated effect for each study^27^. Cochrane’s *χ*^2^ test and the *I*^2^ test were used to evaluate heterogeneity among the studies^28^.

We included the odds ratios of the longest (or shortest) groups in the pairwise meta-analysis (such as long nap vs. no nap, short nap vs. no nap) if the published studies reported odds ratios for various nap times (e.g., 60 to <90 minutes vs. >90 minutes). Stratified analyses were also performed with stratification by study location, study score, and study type, and we used the method of Altman *et al*.^29^ to evaluate whether the pooled ORs differed between groups stratified by the study location. In the study of Stang *et al*.^15^, the prevalence ratio for napping and diabetes was standardized by age, but was not adjusted for other potential confounders. Therefore, we performed a sensitivity analysis that excluded this study and investigated whether there was any change of the point estimate.

Possible publication bias was evaluated by creating a funnel plot of the effect size for each study versus the standard error. Then asymmetry of the funnel plots was assessed by performing Begg’s test^30^ and Egger’s test^31^. To evaluate the potential dose-response relation between diabetes and nap time, a dose-response meta-analysis was performed taking into account the between-study heterogeneity proposed by Orsini *et al*.^32^ to compute the trend from correlated log values of OR estimates across various nap times. A restricted cubic spline model for the duration of nap time with three knots (5th, 35th, 65th, and 95th percentiles)^33^ was estimated by generalized least squares regression analysis, taking into account the correlations within each set of published ORs^34^. Probability (P) values for curve linearity or non-linearity were calculated by testing the null hypothesis that the coefficient of the second spline equals zero. This analysis incorporated data on the ORs and 95% CIs, the number of cases and participants, and the median or mean nap time (minutes per day) for each group.

The midpoint of the upper and lower borders was set as the median dose for each category if the median or mean exposure per category was not reported. If the highest category was open-ended, the midpoint of the category was set at 1.25 times the lower border. For the lowest nap category, we set the median at 0.5 times the cut-off point (e.g., if category was <30 min, the median was set at 15 min). In the study of Lam *et al*.^18^, we set the median time for “napping 1–3 times per week” at 20 min and the median time for “napping 4–6 times per week” at 45 min, taking into account the fact that the mean nap time in this study was 60 min/day.

Because the estimators in the random effects cubic spline model were found to be unstable due to lack of power, we used a fixed effects model to evaluate the dose-response relation. All statistical analyses were performed with Stata V.14.0 software (StataCorp, College Station, Texas, USA). P values of less than 0.05 were considered significant.

This research was carried out according to a predetermined protocol^35^ (Table S2), and it followed the standard guidelines for the conduct and reporting of systematic reviews and network meta-analyses of the Meta-analysis Of Observational Studies in Epidemiology (MOOSE) group^36^ (Checklist S1) and the PRISMA statement^37^ (Checklist S2).

## Results

### Literature search

**Figure 1:**
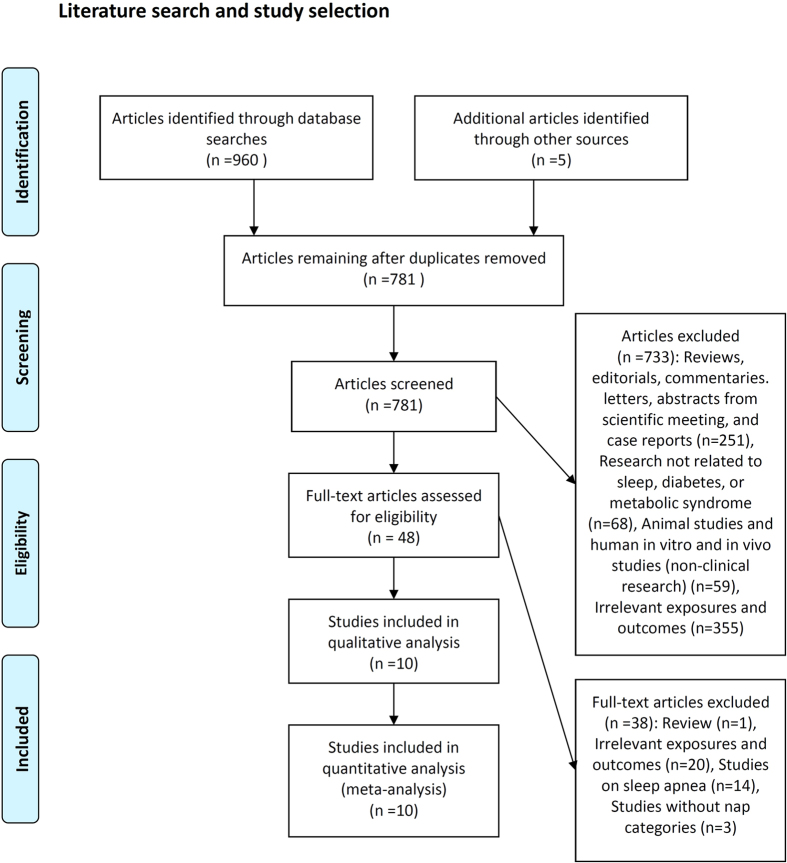
Literature search and study selection.

Figure 1 summarizes the processes employed for literature search and study selection. We identified 965 articles from electronic databases and other sources. After excluding 917 studies that did not meet our inclusion criteria, 48 articles were subjected to full-text evaluation. Finally, 10 studies were included in the meta-analysis^15^,^16^,^17^,^18^,^19^,^20^,^21^,^22^,^23^,^24^. A manual search of the references cited in the reports on these studies did not yield any new eligible articles for assessment. Four of the studies^15^,^19^,^20^,^24^ examined men and women separately, so the data on men and women from each of these studies were handled as if from separate reports. In addition, Lindberg *et al*.^21^ analysed data separately based on the presence of snoring, but we only included the ORs for EDS and diabetes from the EDS without snoring group in the meta-analysis, because the ORs of the two subgroups (EDS with snoring and EDS without snoring subgroups) overlapped. Finally, data on a total of 14 distinct cohorts were included in the meta-analysis.

### Study characteristics

Table 1 lists the characteristics of the studies included in the present analysis, which covered 288,883 subjects from Western and Asian populations, including 20,109 patients with diabetes and 11,222 patients with metabolic syndrome. The mean age, prevalence of napping, and EDS were largely in the range between 60–65 years, 50–70%, and 10–20%, respectively. Four studies were conducted in China^17^,^18^,^19^,^20^, followed by two each in Sweden^21^,^24^, and one each in the USA, Spain, Germany and Finland. In most studies, the analyses were well adjusted for several confounders related to the risk of diabetes and were also adjusted for sleep parameters (such as nocturnal sleep duration). In the study of Lam *et al*.^18^, the estimated relation between a short nap and diabetes was calculated from a subsample (n = 3.822) of the much larger study population (n = 19.567). The definitions of napping and EDS were similar in all of the studies. Study subjects were generally classified as having diabetes if they reported a diagnosis of diabetes made by a physician, were on antidiabetic medication, or had a high fasting plasma glucose level (≥7.0 mmol/L) (Table S3). Subjects were classified as having metabolic syndrome if they fulfilled the criteria of the International Diabetes Federation (IDF)^38^. When the quality of the studies was assessed by using the Newcastle Ottawa Scale, all studies had high scores ≥7 (maximum possible score: 9) (Table S4).

### Results of meta-analysis

#### Napping and type 2 diabetes

**Figure 2:**
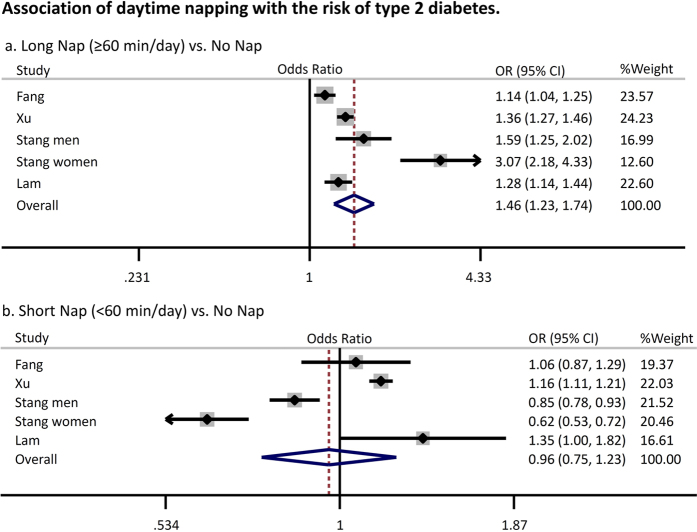
Association of daytime napping with the risk of type 2 diabetes. Plots show the association between daytime napping and the risk of diabetes. CI = confidence interval. OR = odds ratio.

**Figure 3:**
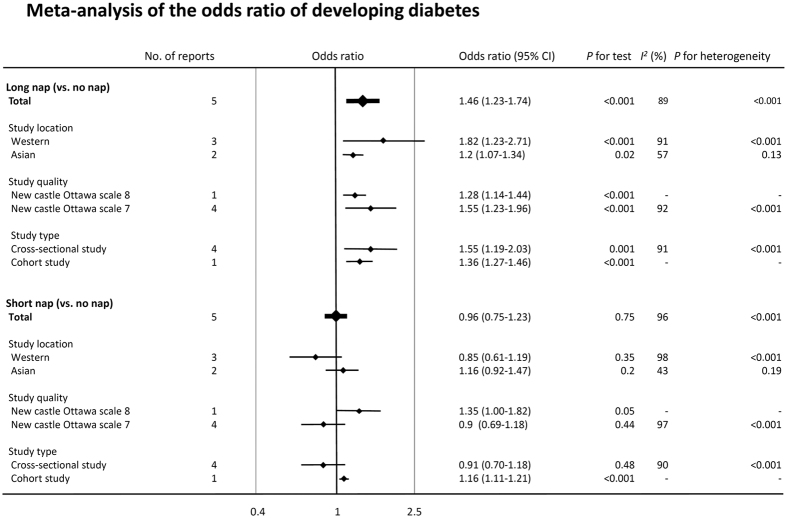
Meta-analysis of the odds ratio of developing diabetes. Plots show the association between daytime napping and the risk of diabetes. CI = confidence interval. OR = odds ratio.

**Figure 4:**
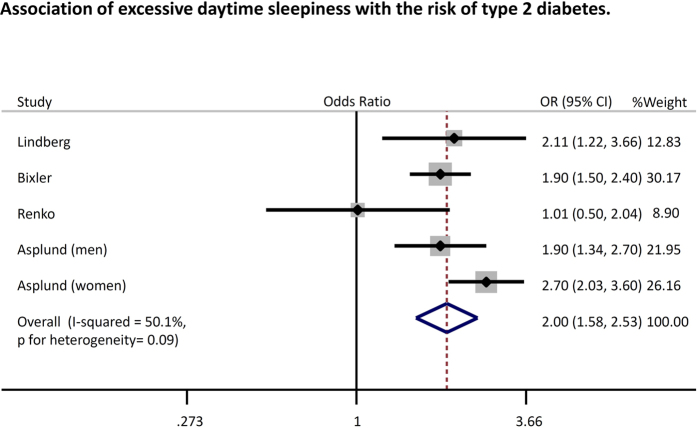
Association of excessive daytime sleepiness with the risk of type 2 diabetes. Plots show the association between excessive daytime sleepiness and the risk of diabetes. CI = confidence interval. OR = odds ratio.

Figure 2 summarizes data on the random effects odds ratio (95% CI) of diabetes. Pooled analysis revealed that a longer nap time (≥60 min/day) was associated with a significantly higher risk of type 2 diabetes (odds ratio: 1.46 [95% confidence interval: 1.23–1.74, *p* < 0.01, *I*^*2*^ = 89%]) compared with the absence of these factors, while a shorter nap time (<60 min/day) was not associated with an increased risk of diabetes (odds ratio 0.96 [0.75–1.23, *p* = 0.75, *I*^*2*^ = 96%]). Similar results were obtained when analyses were performed with stratification by study location, study quality, and study type (Fig. 3). Heterogeneity showed a slight decrease in the analysis stratified by study location (Western or Asian) without any significant interaction (p for interaction = 0.05 for long nap vs. no nap, p for interaction = 0.14 for short nap vs. no nap). Moreover, a sensitivity analysis excluding the study of Stang *et al*.^15^, in which the prevalence ratio for napping and diabetes was standardized by age but not adjusted for other potential confounders, also yielded a similar result (OR1.26 [1.13–1.41, *p* < 0.01, *I*^2^ = 77%) (long nap vs. no nap). The funnel plot, Begg’s test, and Egger’s test did not suggest any evidence of publication bias (Figure S1). In addition, we found that excessive daytime sleepiness was also associated with an increased risk of type 2 diabetes (odds ratio 2.00 [1.58–2.53, *p* < 0.01, *I*^*2*^ = 50%]) (Fig. 4).

**Figure 5:**
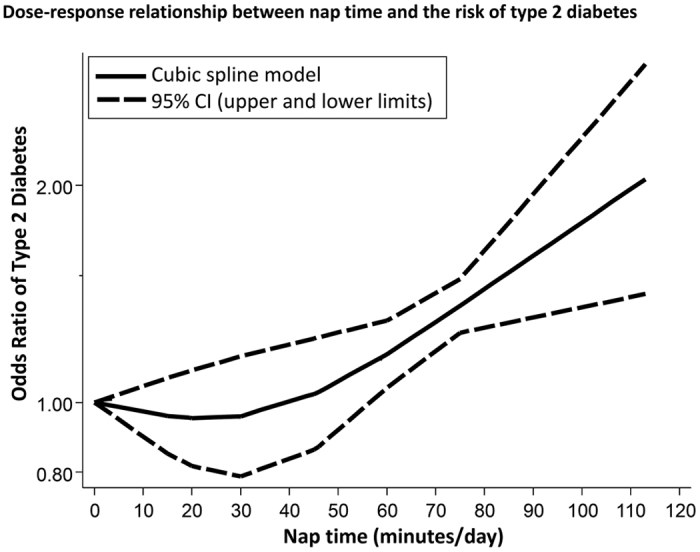
Dose-response relationship between nap time and the risk of type 2 diabetes. CI = confidence interval.

The fixed effects cubic spline model incorporated a total of 208,226 subjects, including 15,571 patients with diabetes. Raw data were available for 5 distinct comparisons with a total of 18 log odds ratios. Cubic spline meta-analysis revealed that there was a J-curve dose-response relation between nap time and the risk of type 2 diabetes (*P* for non-linearity = 0.003) (Fig. 5). That is, the odds ratio of developing type 2 diabetes initially decreased at nap times from 0 to 30 min/day. Then the risk began to increase, but prolongation of the nap time had little effect up to about 40 min/day, followed by a sharp increase in the risk of diabetes at longer times. Napping for 90 minutes increased the risk of developing diabetes by up to 50%.

#### Napping and metabolic syndrome

**Figure 6:**
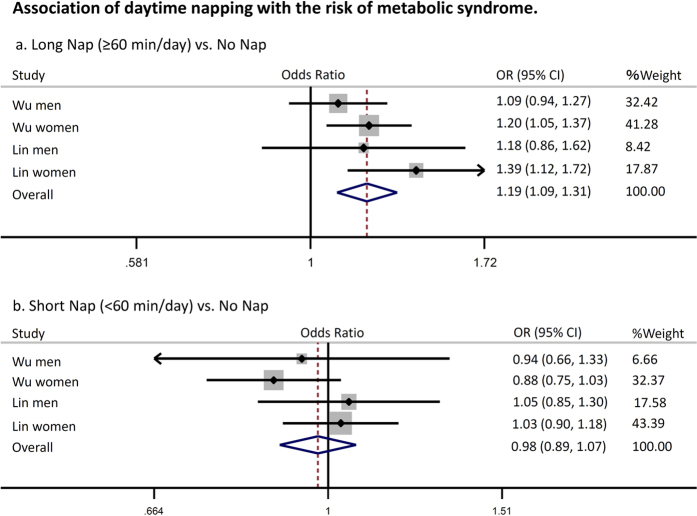
Association of daytime napping with the risk of metabolic syndrome. Plots show the association between daytime napping and the risk of diabetes. CI = confidence interval. OR = odds ratio.

**Figure 7:**
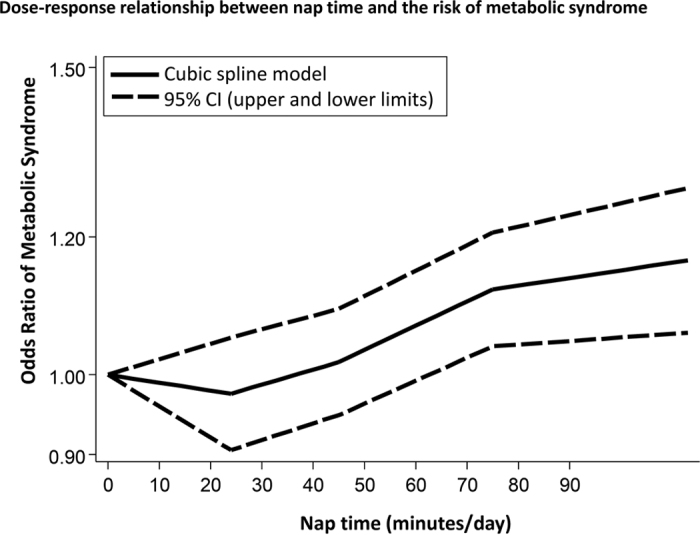
Dose-response relationship between nap time and the risk of metabolic syndrome. CI = confidence interval.

Pooled analysis revealed that a longer nap time (≥60 min/day) was associated with a significantly higher risk of metabolic syndrome (odds ratio: 1.19 [95% confidence interval: 1.09–1.31, *p* < 0.01, *I*^*2*^ = 10%]), while a shorter nap time (<60 min/day) was not associated with an increased risk of metabolic syndrome (odds ratio 0.98 [0.89–1.07, *p* = 0.6, *I*^*2*^ = 0%]) (Fig. 6). Similar to diabetes, the relationship between napping and metabolic syndrome fitted a J-shaped curve (Fig. 7). Napping for less than 40 min/day was not associated with an increased risk of metabolic syndrome, but the risk increased sharply at nap times beyond 40 min. In addition, the funnel plot, Begg’s test, and Egger’s test did not suggest any evidence of publication bias (data not shown).

## Discussion

This meta-analysis showed that either a long nap or EDS was associated with an increased risk of type 2 diabetes and metabolic syndrome compared with not napping or no EDS. In contrast, a short nap was not associated with diabetes or metabolic syndrome. We also demonstrated that nap time may show a J-curve relationship with these metabolic diseases, as has been reported for cardiovascular disease^14^.

As we have stated^14^, it is possible that longer reactive naps are associated with unfavorable outcomes such as poor health, pain, and depression^24^, while older men with sleep apnea may be more likely to take longer naps to compensate for fragmented nocturnal sleep^8^. Several studies have shown that patients with obstructive sleep apnea have increased glucose levels, increased insulin resistance, and a higher risk of type 2 diabetes and metabolic syndrome^24^,^39^,^40^. Although previous studies that involved meta-analysis were well adjusted for the duration of nocturnal sleep (i.e., the quantity of sleep), the quality of sleep was not well adjusted, so the association that we noted between a longer nap time and an increased risk of metabolic diseases might have arisen due to impairment of sleep quality by obstructive sleep apnea or other factors.

Moreover, a shorter nap might not have the same negative effects as a longer nap. Several studies have demonstrated that a daytime nap of less than 30 min promotes wakefulness and alertness, reduces sleep deficits, and enhances performance and learning^41^,^42^,^43^,^44^,^45^. These multiple benefits, especially enhanced athletic performance, might be associated with the lower risk of diabetes for persons taking a short nap in our study. A possible physiological basis for the differing influence of short versus long naps is the relation between nap time and the sleep cycle. A short nap would be expected to end before the onset of deep slow-wave sleep, while a long nap would finish during the slow-wave sleep period. It is known that failing to complete the normal sleep cycle after entering slow-wave sleep can result in a phenomenon known as sleep inertia, which leaves a person feeling groggy, disoriented, and even sleepier than before napping^11^,^46^.

Intermittent hypoxemia and sleep fragmentation increase sympathetic activity^47^, and increased sympathetic activity impairs glucose homeostasis by enhancing the breakdown of glycogen and gluconeogenesis^48^. Alterations of neuroendocrine function and release of proinflammatory mediators (e.g., tumor necrosis factor-α and interleukin-6) may also occur along with autonomic activation^49^,^50^,^51^. Although the mechanisms underlying the beneficial effect of a short nap are still unclear, it might modify these endocrine abnormalities caused by sleep deprivation and improve the circadian rhythm.

Our present meta-analysis had some limitations. First and importantly, while we analysed results from adjusted models because the original studies were observational investigations, this meta-analysis was mainly based on case-control studies and thus cannot prove causal effects. Moreover, the findings could have been influenced by residual confounders or other biases (habits and frailty, health worker bias, fatal malignancy, funding source, etc.). Thus, the associations detected in the individual studies and our meta-analysis may have been biased in either direction. Second, a high level of heterogeneity was detected. To explore the reasons for this, we performed analyses stratified by study location, study quality, and study type. However, heterogeneity was not dramatically reduced by these stratified analyses. Because we identified a nonlinear J-curve relation between nap time and diabetes or metabolic syndrome in our dose-response meta-analysis, it is possible that differences of nap time among the studies analyzed might have led to high heterogeneity. Third, there was limited information about the possible contribution of physical activity to diabetes. Leng *et al*. reported that inactive people have a higher prevalence of napping compared with active people^52^, so the level of activity might be an important confounder.

Ideally, our results should be confirmed by prospective clinical trials, but it seems difficult to perform an interventional longitudinal study of napping in the real world (participants would often be unable to nap for the specified time). Accordingly, we cannot rule out the possibility of “post hoc ergo propter hoc”.

Fourth, we might have overlooked some relevant articles, which could have led to selection bias. However, we investigated all of the references cited in each study we selected to avoid this risk as far as possible. Also, our analyses of publication bias did not suggest that unpublished results had been missed. Fifth, all of the studies used structured interviews or self-completed questionnaires to assess nap times, so measurement error is inevitable. However, measurement error with regard to assessment of exposure is more likely to cause attenuation of a true association than to exaggerate a weak association. Sixth, although our funnel plot analyses did not show significant publication bias, the limited number of studies may have diminished the statistical power for detecting heterogeneity. Finally, we employed different measures (“standardized increment of nap time” or midpoint of nap time) when modeling the associations of the dose-response relation in our meta-analysis. However, none of the studies gave precise data on nap times, which raises concern regarding the precision of our analyses. Despite these limitations, we consider that our meta-analysis provides useful evidence regarding the potential influence of napping on metabolic disease.

In conclusion, the relation between nap time and the risk of diabetes or metabolic syndrome seems to be described by a J-curve, with a sharp increase in the risk of diabetes or metabolic syndrome at longer nap times. In contrast, a short nap was not associated with an increased risk of either diabetes or metabolic syndrome.

Additional research, including large-scale pooling projects, with accurate and detailed measures of napping will be needed to confirm our conclusions and to determine whether adding a nap time score to a conventional risk model improves estimation of the long-term risk of diabetes in the general population, as well as whether a short nap decreases the risk of developing type 2 diabetes.

## Additional Information

**How to cite this article**: Yamada, T. *et al*. J-curve relation between daytime nap duration and type 2 diabetes or metabolic syndrome: A dose-response meta-analysis. *Sci. Rep.* **6**, 38075; doi: 10.1038/srep38075 (2016).

**Publisher's note:** Springer Nature remains neutral with regard to jurisdictional claims in published maps and institutional affiliations.

## Supplementary Material

Supplementary Information

## Figures and Tables

**Table 1:** Summary of cohort studies evaluating the association between napping and type 2 diabetes.

Author, year of publication	Study participants location, subject source, and response rate	Assessment of exposure	Analysis by sex	Prevalence of napping (EDS) (%)
Diabetes and Daytime Napping				
Stang *et al*.^15^ ^a^	Total = 4,458; diabetes = 355 (8.0%); 52% female; age 45–75 years; BMI not available; Germany; Participants were local residents of industrial cities in the Ruhr region of Germany. The response rate was 56%.	Structured interview	Yes	Male 17%, female 15%
Xu *et al*.^16^ ^b^	Total = 174,020; diabetes = 10,100 (5.8%); 43% female; age 62.4 years; BMI 26.5 kg/m^2^; USA; Participants were members of the American Association of Retired Persons from six states and two metropolitan areas of the USA. The response rate was 56%.	Self-completed questionnaire	No	46%
Fang *et al*.^17^^ ^c	Total = 27,009; diabetes = 4,772 (17.7%); 55% female; age 63.6 years; BMI 24.5 kg/m^2^; China; Participants were retired employees of Dongfeng Motor Corporation, an automobile manufacturer in China. The response rate was 87%.	Self-completed questionnaire	No	69%
Lam *et al*.^18^ ^d^	Total = 19,567 (subsample 3.822); diabetes = 2,642 (13.5%); 71% female; age 62.2 years; BMI not available; China; Participants were members of the Guangzhou Health and Happiness Association for Respectable Elders, a community social and welfare association. The response rate was 90% for men and 99% for women.	Structured interview	No	67%
Diabetes and Excessive Daytime Sleepiness				
Lindberg *et al*.^21^^ ^e,^f^	Total = 6,779; diabetes = not available; 100% female; age 44.7 years; BMI 24.1 kg/m^2^; Sweden; Participants were a random sample of women living in the city of Uppsala, Sweden, drawn from the population registry. The response rate was 68.9%.	Self-completed questionnaire	No (female only)	13%
Bixer *et al*.^22^ ^g^	Total = 16,583; diabetes = 2,156 (13%); 74% female; age 46.5 years; BMI 26.3 kg/m^2^; Spain; Participants were a random sample of 16,583 men and women from central Pennsylvania. The response rate was 73.5% (men) and 74.1% (women).	Structured interview	No	8.7%
Renko *et al*.^23^^ ^h	Total = 593; diabetes = 84 (14.3%); 59% female; age 60 years; BMI not available; Finland; Participants were living in the City of Oulu in northern Finland. The response rate was 71%.	Self-completed questionnaire	No	19.6%
Asplund.^24^ ^i^	Total = 6,143; diabetes = not available; 61% female; age 73 years; BMI not available; Sweden; Participants were members of the National Swedish Pensioners’ Association. The response rate was 61%.	Self-completed questionnaire	Yes	Male 14%, female 14%
Metabolic Syndrome and Daytime Napping				
Wu *et al*.^19^^ ^j	Total = 25,184; metabolic syndrome = 8,046 (31.9%); 55% female; age 63.6 years; BMI 25.8 kg/m^2^; China; Participants were retired employees of Dongfeng Motor Corporation in Shiyan City, Hubei Province, China. The response rate was 87%.	Self-completed questionnaire	Yes	Male 73%, female 65%
Lin *et al*.^20^ ^k^	Total = 8,547; metabolic syndrome = 3,176 (37.2%); 28.2% female; age 56.0 years; BMI 23.7 kg/m^2^; China; Participants were from a community in Guangzhou, China. The response rate was 98.1%.	Self-completed questionnaire	Yes	Male 56%, female 48%

Among these studies, one study (16) was a cohort study with a mean follow-up period of 7–10 years. All of the other studies were cross-sectional studies.

^a^Analysis was adjusted for age, hypertension, smoking status, dyslipidemia, BMI, waist circumstance, CRP, Agatston score, and ABI.

^b^Analysis was adjusted for age, sex, race, education, marital status, smoking, coffee and alcohol consumption, calorie intake, family history of diabetes, general health status, nocturnal sleep duration, physical activity, and BMI.

^c^Analysis was adjusted for age, sex, marriage, education, smoking status, alcohol intake, hypertension, coronary heart disease, stroke, nocturnal sleep duration, BMI, and physical activity.

^d^Analysis was adjusted for age, sex, educational level, occupation, smoking, alcohol intake, physical activity, health status (self-rated health, hospitalization, hypertension, cardiovascular disease, and family history of diabetes), adiposity and metabolic markers (waist circumference, triglycerides, and total cholesterol), and sleep variables (total sleep duration, insomnia, daytime sleepiness, and snoring). The estimate for the relation between a short nap time and diabetes was calculated from a subsample (n = 3,822) of the much larger study population (n = 19,567).

^e^Analysis was stratified by presence of snoring.

^f^Analysis was adjusted for age, BMI, alcohol dependency, level of physical activity, and smoking status.

^g^Analysis was adjusted for age, sex, BMI, depression, smoking, alcohol use, allergy, asthma, hypertension, thyroid function, night time sleep duration, and objective polysomnographic data.

^h^Analysis was adjusted for sex, depression, use of sleeping medication, smoking, and BMI.

^i^Analysis was adjusted for age, general health, frequent awakening, ability to fall asleep again after nocturnal awakening, and hypnotic medication.

^j^Analysis was adjusted for age, marriage, education, smoking status, drinking status, physical activity, coronary heart disease, myocardial infarction, stroke, family history of hypertension and diabetes, BMI, and nighttime sleep duration.

^k^Analysis was adjusted for age, BMI, current smoking, drinking status, physical activity, and sleeping hours (total nocturnal sleeping hours).
