# Supplementary material for: J-curve relation between daytime nap duration and type 2 diabetes or metabolic syndrome: A dose-response meta-analysis
Source: Sci Rep. 2016 Dec 2;6:38075. doi: 10.1038/srep38075 (PMC5133463; doi:10.1038/srep38075)

J-curve relation between daytime nap duration and type 2 diabetes or metabolic syndrome: A dose-response meta-analysis

Tomohide Yamada, MD, PhD

Nobuhiro Shojima, MD, PhD

Toshimasa Yamauchi, MD, PhD

Takashi Kadowaki, MD, PhD

Department of Diabetes and Metabolic Diseases, Graduate School of Medicine

University of Tokyo, Japan

Supplementary information

Table S1. Search terms used for the electronic databases

Table S2. Protocol for Systematic Review

Table S3. Definitions of napping and diabetes in each study

Table S4. New castle-Ottawa Scale score of each study

Figure S1. Funnel plots and the results of Begg's test and Egger's test

Table S1. Search terms used for the electronic databases

Database	Search terms
Medline N=370	(("diabetes mellitus"[MeSH Terms] OR ("diabetes"[All Fields] AND "mellitus"[All Fields]) OR "diabetes mellitus"[All Fields] OR "diabetes"[All Fields] OR "diabetes insipidus"[MeSH Terms] OR ("diabetes"[All Fields] AND "insipidus"[All Fields]) OR "diabetes insipidus"[All Fields]) OR (metabolic[All Fields] AND ("syndrome"[MeSH Terms] OR "syndrome"[All Fields]))) AND (nap[All Fields] OR siesta[All Fields] OR (daytime[All Fields] AND ("sleep stages"[MeSH Terms] OR ("sleep"[All Fields] AND "stages"[All Fields]) OR "sleep stages"[All Fields] OR "sleepiness"[All Fields])))
Web of Science N=560	((diabetes OR metabolic syndrome) AND (nap OR siesta OR daytime sleepiness)) Limit: Document type=(ARTICLE)
Cochrane Library N=30	(diabetes OR glucose OR dysglycemia) AND (nap OR siesta OR daytime sleepiness)

Table S2. PROTOCOL FOR SYSTEMATIC REVIEW

J-curve relation between daytime nap duration and type 2 diabetes or metabolic syndrome: A dose-response meta-analysis

Information	Topic	Date	PRISMA-P Item*
**ADMINISTRATIVE INFORMATION**			
**Title**			
J-curve relation between daytime nap duration and type 2 diabetes or metabolic syndrome: A dose-response meta-analysis	Identification	Jan 2016	1a
**Registration**			
NA	-	Jan 2016	2
**Authors**			
Tomohide Yamada (bqx07367@yahoo.co.jp)	Contact	Jan 2016	3a
Nobuhiro Shojima			
Toshimasa Yamauchi			
Takashi Kadowaki			
All Authors contributed to this protocol. T Yamada is a guarantor of the review.	Contribution		3b
**Amendments**			
NA (This is not an amendment of a previously completed/published protocol)	-	Jan 2016	4
**Support**			
T Yamada was funded by Japan Diabetes Society, Banyu Foundation, KAKENHI (Grants-in-Aid for Scientific Research), Japan Foundation for Applied Enzymology, and Japan Association for Diabetes Education and Care. We declare that these funds have not influenced this research.	Sources	Jan 2016	5a
NA	Sponsor		5b

The funding sources had no role in this study.	Role of sponsor/funder		5c
**INTRODUCTION**			
**Rationale**			
Adequate sleep is important for good health, but it is not always easy to achieve because of social factors. Daytime napping is widely prevalent around the world. Recent epidemiological studies on the relation between daytime napping and diabetes or metabolic syndrome have yielded conflicting results	-	Jan 2016	6
**Objectives**			
To investigate the association between napping or excessive daytime sleepiness and the risk of diabetes or metabolic syndrome, and to quantify the potential dose-response relation by using cubic spline models.	-	Jan 2016	7
**METHODS**			
**Eligibility criteria**			
Observational studies that reported risk estimates for type 2 diabetes and metabolic syndrome in relation to daytime napping and excessive daytime sleepiness in the general population, and that provided point estimates of odds ratio with the 95% confidence interval or standard error for qualitative assessment.	-	Jan 2016	8

**Information sources**			
Medline, ISI Web of Science, Cochrane Library; Study Authors	-	Jan 2016	9
**Search Strategy**			
{PubMed} (“Diabetes” OR “Metabolic syndrome”) AND (“Nap” OR “Siesta” OR “Daytime sleepiness”)	-	Jan 2016	10

**Study Records**			
Two authors (TYamada, NS) will independently perform the searches. Literature search results will be uploaded to EndNote	Data management	Jan 2016	11a
Two authors (TYamada, NS) will independently screen titles/abstracts and obtain full reports for 1) reports meeting inclusion criteria; 2) those requiring further discussion. Any discrepancies will be resolved through discussion.	Selection Process	Jan 2016	11b
Extracted data will be independently (TYamada, NS) add into digital pre-defined forms (Excel).	Data collection process	Jan 2016	11c
**Data Items**			
We extract information on the characteristics of each study (study name, authors, year of publication, journal, study type, study location, and number of participants and incident cases), the subject characteristics (age, sex, and BMI), the extent of exposure to napping (definition of napping, nap time, and prevalence of napping in each category), the validity of the method used for assessment of napping (and excessive daytime sleepiness), the validity of the method used for assessment of the outcome (diabetes and metabolic syndrome), and the validity of the analytical methods (statistical models, covariates included in the models, and risk estimates for each nap duration category).	-	Jan 2016	12
**Outcomes and Prioritization**			
The odds ratio (OR) and its 95% confidence interval (CI) will be employed as the measure of association in all studies	-	Jan 2016	13
**Risk of Bias in Individual Studies**			
Study-level quality will be assessed using the Newcastle Ottawa Scale by two authors (TYamada, NS) and disagreement will be resolved through discussion.	-	Jan 2016	14
**Data Synthesis**			

We will conduct a meta-analysis for each outcome using the DerSimonian-Laird random effects model to compare napping categories and set study weights as equal to the inverse variance of the estimated effect for each study. To evaluate the potential dose-response relation between diabetes and nap time, a dose-response meta-analysis will be performed taking into account the between-study heterogeneity proposed by Orsini et al. to compute the trend from correlated log values of OR estimates across various nap times. A restricted cubic spline model for the duration of nap time with three knots (5th, 35th, 65th, and 95th percentiles) will be estimated by generalized least squares regression analysis, taking into account the correlations within each set of published ORs. Probability (P) values for curve linearity or non-linearity will be calculated by testing the null hypothesis that the coefficient of the second spline equals zero. This analysis will incorporate data on the ORs and 95% CIs, the number of cases and participants, and the median or mean nap time (minutes per day) for each group. The midpoint of the upper and lower borders will be set as the median dose for each category if the median or mean exposure per category was not reported. If the highest category is open-ended, the midpoint of the category was set at 1.25 times the lower border. For the lowest nap category, we set the median at 0.5 times the cut-off point (e.g., if category was <30 min, the median was set at 15 min).	-	Jan 2016	15a
Cochrane's I2 test and the I2 test will be used to evaluate heterogeneity among the studies. Stratified analyses will be also performed (with stratification by study location, study score, and study type). Possible publication bias will be evaluated by creating a funnel plot of the effect size for each study versus the standard error. Then asymmetry of the funnel plots will be was assessed by performing Begg's test and Egger's test.	-	Jan 2016	15b
Additional analyses: NA	-	Jan 2016	15c
Summary planned if quantitative synthesis not appropriate: NA	-	Jan 2016	15d
**Meta-bias(es)**			
Tables will show the availability of data for each study and outcome (selective reporting)	–	Jan 2016	16
**Confidence in cumulative evidence**			
Results will be commented in view of study limitations and available evidence	-	Jan 2016	17

Shamseer L, Moher D, Clarke M, Ghersi D, Liberati A, Petticrew M, et al. PRISMA-P Group. Preferred reporting items for systematic review and meta-analysis protocols (PRISMA-P) 2015: elaboration and explanation. BMJ. 2015 Jan 2;349:g7647. doi: 10.1136/bmj.g7647.

Table S3. Definitions of napping and diabetes in each study

Diabetes and Daytime Napping

Author, Year	Definition of napping	Definition of diabetes
Stang et al, 2007 (15)	Participants were asked to report whether they were taking a midday nap, as well as its frequency and duration.	Participants were classified as having diabetes if they reported a history of diagnosis of the disease, took antidiabetic drugs, or had a non-fasting glucose level ≥200 mg/dl.
Xu et al, 2010 (16)	Participants were asked to report whether they were taking a midday nap, as well as its frequency and duration.	Participants were asked whether they had ever been told that they had diabetes by a doctor.
Fang et al, 2013 (17)	Participants were asked to report whether they were taking a midday nap, as well as its frequency and duration.	Participants were classified as having diabetes if they reported a history of diagnosis of diabetes by a physician, were on antidiabetic medication, or had a high fasting plasma glucose level (≥7.0 mmol/L).
Lam et al, 2010 (18)	Participants were asked to report whether they were taking a midday nap, as well as its frequency and duration.	Participants were classified as having diabetes if they reported a history of diagnosis of diabetes by a physician, were on antidiabetic medication, or had a high fasting plasma glucose level (≥7.0 mmol/L).

Diabetes and Excessive Daytime Sleepiness

Author, Year	Definition of EDS	Definition of diabetes
Lindberg et al. 2007 (21)	Participants were asked to report whether they fell asleep involuntarily for a short period during the day, e.g., when there was a pause at work.	Participants were asked whether they had ever been told that they had diabetes by a doctor.
Bixer et al. 2005 (22)	Participants were asked to report whether they felt drowsy or sleepy most of the day, but manage to stay awake.	Participants were classified as having diabetes if they had current treatment for diabetes or a fasting blood glucose level >126 mg/dl.
Renko et al. 2005 (23)	Participants were asked to report whether they noted sleepiness in the daytime.	Participants were classified as having diabetes if they fulfilled the criteria for diabetes in the75g OGTT.
Asplund. 1995 (24)	Participants were asked to report whether they were often sleepy in the daytime.	Participants were asked whether they had ever been told that they had diabetes by a doctor.

Metabolic Syndrome and Daytime Napping

Author, Year	Definition of napping	Definition of metabolic syndrome
Wu et al. 2015 (19)	Participants were asked to report whether they had a habit of taking a nap after lunch.	Participants were classified as having metabolic syndrome if they fulfilled the criteria of the International Diabetes Federation [Lancet 2005;366(9491):1059-62].
Lin et al. 2014 (20)	Participants were asked to report whether they had habitually napped in the daytime every day during the past seven days.	Participants were classified as having metabolic syndrome if they fulfilled the criteria of the International Diabetes Federation [Lancet 2005;366(9491):1059-62].

Table S4. New castle-Ottawa Scale score of each study

Cohort study

Author, Year	Quality Score	Selection				Comparability of Cohorts		Outcome		
		Representativeness of Exposed Cohort	Representativeness of Non-exposed Cohort	Ascertainment of Exposure	Outcome Not Present at Beginning of Study	Control for age	Control for sleep duration and/or sex	Assessment of Outcome	Was Follow-up Long Enough?	Adequacy of Follow-Up
Xu, 2010 (16)	7	*	*	Self-reported	*	*	*	Self-reported	*	*

Non-cohort study

Author, Year	Quality Score	Selection				Comparability of Cases and Control		Exposure		
		Is the case definition Adequate?	Representativeness of the cases	Selection of controls	Definition of Control	Control for age	Control for sleep duration and/or sex	Assessment of Exposure	Same method of ascertainment for cases and controls	Non-Response rate
Stang, 2007 (15)	7	Self-reported	*	*	*	*	*	*	*	Not described
Fang, 2013 (17)	7	Self-reported	*	*	*	*	*	*	*	Not described
Lam, 2010 (18)	8	*	*	*	*	*	*	*	*	Not described
Lindberg, 2007 (21)	7	Self-reported	*	*	*	*	*	*	*	Not described
Bixer, 2005 (22)	7	Self-reported	*	*	*	*	*	*	*	Not described
Renko, 2005 (23)	8	*	*	*	*	*	*	*	*	Not described
Asplund, 1995 (24)	7	Self-reported	*	*	*	*	*	*	*	Not described
Wu et al. 2015 (19)	8	*	*	*	*	*	*	*	*	Not described
Lin et al. 2014 (20)	8	*	*	*	*	*	*	*	*	Not described

Figure S1. Funnel plots and the results of Begg's test and Egger's test

Long nap vs. no nap

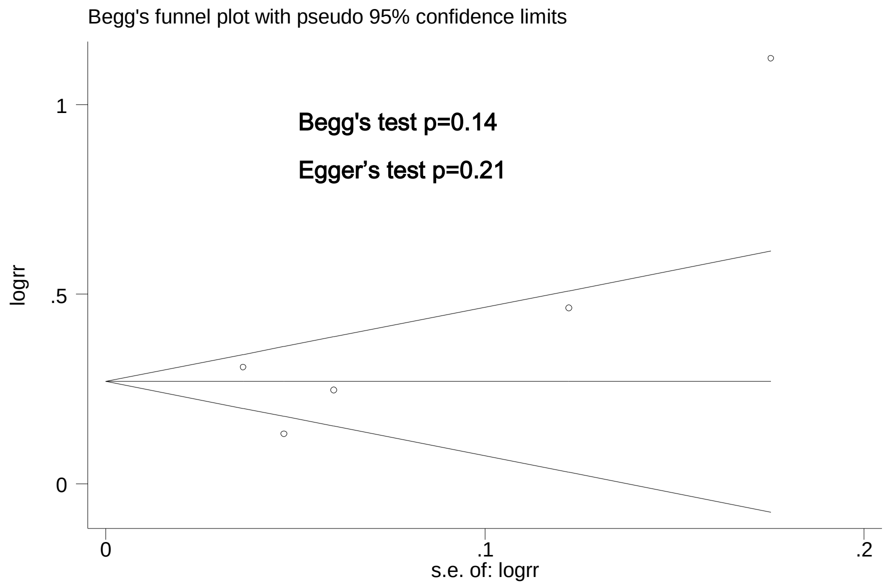

Short nap vs. no nap

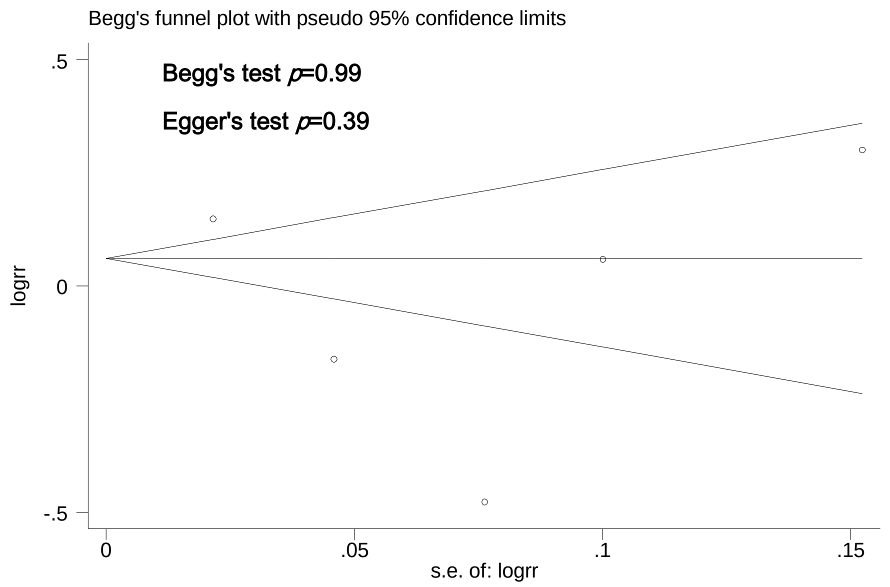

Supplement: Supplementary Information [file srep38075-s1.pdf]
